# How Does Gender Moderate Customer Intention of Shopping via Live-Streaming Apps during the COVID-19 Pandemic Lockdown Period?

**DOI:** 10.3390/ijerph182413004

**Published:** 2021-12-09

**Authors:** Yuyang Zhao, Fernando Bacao

**Affiliations:** NOVA Information Management School (NOVA IMS), Universidade Nova de Lisboa, 1070-312 Lisboa, Portugal; bacao@novaims.unl.pt

**Keywords:** COVID-19, customer behavior, psychological process, live-streaming shopping apps, stimulus-organism-response framework, Unified Theory of Acceptance and Use of Technology 2, Flow Theory, gender, age

## Abstract

Shopping through Live-Streaming Shopping Apps (LSSAs) as an emerging consumption phenomenon has increased dramatically in recent years, especially during the COVID-19 lockdown period. However, insufficient studies have focused on the psychological processes undergone in different customer demographics while shopping via LSSAs under pandemic conditions. This study integrated the Unified Theory of Acceptance and Use of Technology 2 with Flow Theory into a Stimulus-Organism-Response framework to investigate the psychological processes of different customer demographics during the COVID-19 lockdown period. A total of 374 validated data were analyzed by covariance-based structural equation modelling. The statistical results demonstrated by the proposed model showed a significant discrepancy between different gender groups, in which Flow, as a mediator, representing users’ engagement and immersion in shopping via LSSAs, was significantly moderated by gender where connection between stimulus components, hedonic motivation, trust and social influence and response component perceived value are concerned. This study contributed a theoretical development and a practical framework to the explanation of the mental processes of different customer demographics when using an innovative e-commerce technology. Furthermore, the results can support the relevant stakeholders in e-commerce in their comprehensive understanding of customers’ behavior, allowing better strategical and managerial development.

## 1. Introduction

Live-streaming commerce, as a burgeoning e-commerce pattern with the unique features of real-time live-streaming demonstration of products and instant interactions among sellers and viewers, provides personalized services for customers remotely [1]. Meanwhile, based on the broad application of telecommunication networks and extensive adoption of mobile devices, live-streaming shopping apps (LSSAs) have provided an immersive experience for viewers [2], which has formulated a new consumption phenomenon, namely, shopping via LSSAs, especially in the Chinese e-commerce industry in recent years. According to a report from iiMedia (2020), live-streaming commerce industry transactions were estimated to exceed 129 billion USD in 2020, up from 61 billion USD in 2019 [3]. In 2019, on the Taobao e-commerce platform alone, over 60,000 live-streaming shows hosted by brands, stores and celebrities attracted more than 400 million consumers [4]. Shopping via LSSAs has established an entertainment environment for customers, facilitating a revolution in commerce. Under the lockdown measures for the defence against COVID-19 transmission especially, interaction and entertainment were in significant demand from individuals. Despite several previous studies demonstrating that the adoption of LSSAs was influenced by the motivations of participants [5,6], technology features [7] and human-computer interaction [2], few studies have focused on the customer’s psychological processes when subject to the moderating effects of age and gender in a particular environment. LSSAs are mobile entertainment and commerce applications, and their adoption should take the moderating effects of age and gender into consideration [8].

Consequently, the objective of the current study is to investigate the psychological processes of customers of different ages and genders when shopping via LSSAs during the COVID-19 pandemic lockdown situation. The proposed model embeds the revised Unified Theory of Acceptance and Use of Technology 2 (UTAUT2) as a stimulus, along with Flow Theory as an organism, into the stimulus-organism-response (SOR) framework. In this way, the SOR framework, as the main structural foundation of the research model, explains that customers’ behavioral psychological processes are determined by external antecedents as well as internal cognitions [9,10,11,12]. UTAUT2, as a theoretical framework, has coordinated consumer-oriented perceptions to predict users’ behavioral intentions [13], which is legitimately considered a perceptive process in the explanation of customers’ perceptions of LSSAs and the constitution of stimulus components. Flow Theory supports organisms theoretically by representing participants’ concentration and engagement in shopping activities via LSSAs [14]. These theoretical frameworks are initially integrated and verified in this study, supporting relevant researchers and stakeholders in order to better understand the behaviors of customers.

The current study comprises eight sections, investigating the psychological processes of customers shopping via LSSAs under pandemic lockdown conditions. Section 2 consists of a literature review regarding the subject of shopping via LSSAs, as well as relevant theoretical frameworks. This section is followed by the research model and hypotheses development, which are presented in Section 3. Section 4 illustrates the method of data collection and the demographic distribution of data. Subsequently, Section 5 presents the results of the data analysis. Section 6 discusses the findings of these results, while Section 7 illustrates their theoretical and practical implications. Finally, limitations and recommendations for future research and conclusion are outlined in Section 8.

## 2. Theoretical Background

### 2.1. Live-Streaming Shopping Apps (LSSAs)

The current LSSAs comprise e-commerce functions that are integrated into live-streaming platforms with simultaneous and authentic consumption interactions between vendors and customers [5,6]. Shopping via LSSAs can be divided into two patterns. The first of these patterns is consumption activities on mobile e-commerce apps with extensional live-streaming functions, such as Taobao and AliExpress, while the second is shopping via LSSAs through a third-party e-commerce service, such as Tiktok and LiveMe [6]. Shopping via LSSAs has become a thriving new consumption phenomenon. Off-line consumption activities were restricted, especially under lockdown conditions during the COVID-19 pandemic; shopping via LSSAs supported customers’ daily supply and demand requirements and provided a relaxation pattern during the quarantine time, formulating a positive perception among users. Cai et al. (2018) claimed that customers’ decisions to shop via live-streaming are not only influenced by utilitarian perceptions of service and production but are also determined by hedonic motivation [5]. According to the entertainment feature of live-streaming, viewers’ engagement and gratification significantly affected their shopping activities [15]. Based on LSSA’s facilitation of conspicuous human-machine interaction, this affordance of LSSAs and customers’ engagement conjointly determine the number of customers purchasing via LSSAs [2].

Meanwhile, previous studies have found that customers’ endorsement of, and behavioral responses to LSSAs were observably determined by their intrinsic and extrinsic motivations, social influence, entertainment, perceived flow and emotional engagement [16,17,18]. Accordingly, customers’ mental perceptions, such as trust and perceived value, have significantly influenced their engagement with live-streaming commerce [1]. Moreover, the simultaneity, authenticity, interactivity and customizability characteristics of LSSAs significantly formulate customers’ perceptions of this technology, affecting their behavior [7,19]. However, prior literature has insufficiently investigated customers’ psychological processes while shopping via LSSAs under specific conditions. Moreover, the moderating effects of age and gender might lead to different results in different market segmentations [20]. Thus, it is meritorious to clarify the role of age and gender in moderating customers’ mental processes while shopping via LSSAs in the pandemic lockdown situation.

### 2.2. Stimulus-Organism-Response (SOR) Framework

The SOR framework demonstrates that external antecedents influence customers’ psychological processing, first as a perceptive stimulus, affecting their cognitive and emotional reflections, then as an organism, contributing towards formulating their mental or behavioral traits, and finally as a response, such as an attitude, adoption intention or actual usage [21]. The SOR framework has been modified with external variables to analyze in a qualified way the connections between the stimulus (environmental input), the organism (mental process) and the response (behavioral outputs), in order to explain users’ behaviors in various business analysis studies [11,22] as well as works of literature on the adoption of innovative technology [10,12,23], which are demonstrated in Table 1. In their investigation of users’ visiting intentions in virtual reality tourism, Kim, Lee and Jung (2020) designated customers’ actual experiences as stimuli, their cognition and affection (including enjoyment, emotional involvement and flow) as organisms and their attachment and intention as responses [12]. Zhao, Wang and Sun (2020) proposed that stimuli include interactivity, media richness and sociability, and assumed that virtual experience as an organism would include telepresence, social presence and flow, which in turn determined students’ continuing intentions regarding the use of massive open online courses [23]. Moreover, the SOR framework has been applied in an investigation of customers’ online shopping intentions, which were significantly influenced by their attitude, which was in turn affected by their internal and external environment [9]. However, compared with traditional online shopping, streaming service quality and promotion campaigns played more significant roles in formulating customers’ purchase intentions via mobile shopping apps [22].

Furthermore, the moderating effects of age and gender have rarely been examined in the SOR framework. There have been a few previous studies partially involving the moderators in the SOR model, but their results were presented inconsistently. Wu and Li (2018) found that gender had a significant moderating effect on customers’ loyalty in online social commerce [11], against the findings of Islam and Rahman (2017) [24]. Therefore, this study uses the SOR framework as a theoretical foundation to create a research model.

### 2.3. Unified Theory of Acceptance and Use of Technology 2 (UTAUT2)

UTAUT2 was developed by Venkatesh, Thong and Xu (2012). As an extension of the UTAUT model, UTAUT2 predicts users’ technological perceptions, determining their intention regarding the adoption of a particular technology [13]. Several researchers have modified UTAUT2 in miscellaneous mobile-technology-adoption studies by extending it with additional variables or moderators, for example, trust [25,26], privacy [27] and Hofstede’s cultural values [28,29]. Some studies have incorporated UTAUT2 into other theoretical frameworks, such as diffusion of innovation [30] and the expectation-confirmation model [31,32], in investigating customers’ behaviors while using mobile technology. Furthermore, UTAUT2 involves the use of age, gender and experience as moderators to explain individual differences in adoption intention [13]. Moreover, UTAUT2 was applied to the adoption of mobile shopping applications by Tak and Panwar (2017), who found that hedonic motivation was the most significant antecedent, which corresponds with the recognition of the current study that LSSAs are entertaining mobile shopping applications [33]. Therefore, UTAUT2 is considered the appropriate theoretical foundation for investigating users’ perceptions as a stimulus in the proposed model.

### 2.4. Flow Theory

Flow Theory was initially proposed by Csikszentmihalyi (1975) as a way to predict individuals’ mental engagement in a certain activity [14]. Subsequently, Flow Theory’s applicability has been extended into the human-computer interaction domain to describe users’ absorption in technology [34]. Specifically, flow represents users’ holistic, immersive consciousness when they concentrate entirely on a particular activity or technology; their involvement will be self-reinforced by constitutional enjoyment and engaging interactivity, and, in turn, their self-consciousness will become indistinct in order to ignore irrelevant interruptions [35]. Flow has been applied as a mediator in various technology adoption studies to describe customers’ cognition and engagement for predicting users’ adoption intention [10,36,37], especially in the fields of entertaining technologies, such as live-streaming [16] and mobile shopping [38]. Flow is significantly influenced by users’ technological perceptions [37,39], as well as mental determinants such as emotion [10], trust [38] and enjoyment [16]. Meanwhile, the combination of Flow Theory with other frameworks, such as the Information Systems Success Model [37,38] and the Stimulus-Organism-Response framework [10], also reasonably illustrated users’ adoption intention. Thus, Flow Theory is considered a theoretical foundation for the representation of users’ shopping engagement via LSSAs during the pandemic lockdown period, acting as the organism in the proposed model.

### 2.5. Moderating Effects of Age and Gender

According to the current research objectives, age and gender are proposed as moderating variables involved in the analysis process. Venkatesh, Thong and Xu (2012) initially confirmed that age and gender have moderating effects on the UTAUT2 constructs affecting users’ adoption intention [13]. Moreover, other works of literature have integrated age and gender as moderators within various frameworks (UTAUT, Technology Acceptance Model (TAM), Diffusion of Innovation (DOI) Theory) and have confirmed that age and gender significantly moderate constructs in different contexts [20,40,41,42,43,44]. However, based on the differences in research objectives, sample targets and involved variables in various scenarios, the moderating effects of age and gender have been diverse in different literature. Venkatesh and Zhang (2010) validated the idea that performance expectancy in the behavioral intention of information technology was significantly moderated by younger male users, and effort expectancy was strongly moderated by older female customers [40], which is contrary to the findings of Riskinanto, Kelana and Hilmawan (2017), who claimed that stated age had insignificant effects on perceived usefulness and ease of use regarding the intention of adoption of E-payment technology [43]. On the other hand, Liébana-Cabanillas, Sánchez-Fernández and Muñoz-Leiva (2014) complementarily illustrated that social influence bore a strong influence on users above 35 years old, and that trust was more affected by younger groups in the adoption of mobile payments [41]. Moreover, Shao et al. (2018) claimed that males had a stronger moderating effect on mobility and reputation in the trust-formation process of mobile payments, while females moderated customization and security of trust more [44]. Likewise, Pascual-Miguel, Agudo-Peregrina and Chaparro-Peláez (2015) found that the moderating effects of female customers on effort expectancy and social influence were significantly stronger than male customers on online purchase intention [45]. In order to analyse the moderating effects of age and gender on all constructs in the proposed research model, a multi-group analysis is applied in this research, which is widely applied in previous studies for multi-group comparisons [20,44,45].

## 3. Development of Research Model and Hypotheses

**Figure 1:**
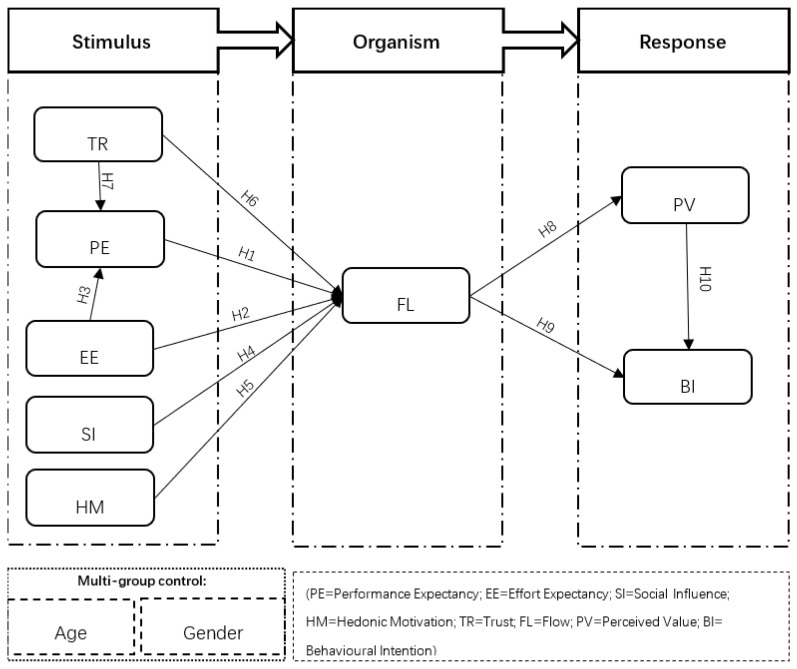
Proposed research model.

Based on the previous literature reviews, the integration of the SOR framework with UTAUT2 and Flow Theory is considered a theoretical foundation on which to propose a comprehensive model for the investigation. Specifically, according to previous paradigms of the SOR framework application, this research extends the SOR framework by integrating variables from the revised UTAUT2 model, which are proposed as stimulus components, roused by technological perceptions of LSSAs during the pandemic lockdown period (performance expectancy, effort expectance) [22,24], namely, social influence [11], hedonic motivation [12] and trust [12]. These variables reflect users’ external and internal perceptions towards inciting their further psychological cognition. On the other hand, due to the popularization of smartphones, proficiency in using various mobile applications and the absence of monetary cost in the operation of LSSAs, original variables such as facilitating conditions, habit and price value are excluded from the UTAUT2 model, which is in accordance with previous findings [25,26,28,31,32]. Flow Theory provides theoretical support to the reflection of customers’ mental, cognitive and affective intermediary states during shopping via LSSAs in the COVID-19 pandemic lockdown period, which it is appropriate to consider as an organism in the SOR framework [10,12,23]. Moreover, this study proposes that perceived value and adoption intention reflects customers’ psychological reactions and behaviors, constituting the response elements of the SOR framework [10,12]. In addition, age and gender are considered moderators of the theoretical model comparing the different effects of antecedents on customers’ adoption intention regarding LSSAs in each subgroup. The proposed research model is generalized and presented in Figure 1 with the relevant hypotheses relations.

### 3.1. Stimulus Components: Variables from the Revised UTAUT2 Model

Performance expectancy (PE), as a technological perception, represents the perceived usefulness of a certain technology in the eyes of a user, and its potential to optimize their experience of a specific technology or to reinforce their performance in particular activities [13]. Moreover, the technological features which users perceive, such as compatibility, service quality, information quality and system quality, can be generalized as perceived usability of technology, represented as PE [37,46,47]. Related to technology adoption, PE significantly formulates users’ mental responses, such as attitude, adoption intention and continued usage intention, which are confirmed by prior literature [30,48,49]. Accordingly, customers’ psychological cognitions are formulated by their perceptions of satisfying usability, which indicates that PE significantly influences customers’ perceived flow when they intend to adopt new technology [10,39]. Therefore, the hypothesis can be generalized as follows:

**Hypothesis** **1** **(H1).** *Customers’ performance expectancy (PE) as a stimulus positively determines the organism flow (FL) when shopping via LSSAs during the pandemic lockdown period.*

Effort expectancy (EE), as a technological perception, expresses the idea that users acquire feelings of easiness from understanding, operating and interacting with a specific information technology [13]. A variety of literature has verified EE’s considerable effect on customers’ attitude and behavioral intention in technology adoption research [43,47,48]. Consequently, customers’ engagement and flow experience are formulated by understandability, operability and intractability [36,39]. Moreover, the influence of EE has been confirmed by Kim et al. (2013) as not only affecting flow but also performance expectancy when users adopt entertainment technology [39]. When customers recognize that a technology is easy to access, they will tend to confirm its usability. This phenomenon has been validated in various technology adoption works of literature, such as live-streaming [19], mobile banking [26,46,49] and mobile payment [41,47]. Thus, the hypotheses related to EE are proposed as follows:

**Hypothesis** **2** **(H2).** *Customers’ effort expectancy (EE) as a stimulus positively determines the organism flow (FL) when shopping via LSSAs during the pandemic lockdown period.*

**Hypothesis** **3** **(H3).** *Customers’ effort expectancy (EE) as a stimulus positively determines performance expectancy (PE) when shopping via LSSAs during the pandemic lockdown period.*

Social influence (SI), as an environmental perception, represents customers perceiving an influence from people particularly relevant to them, such as close friends, family members and colleagues, who recommend and support them in using a certain technology [13]. Customers’ anxiety is derived from the uncertainty of new technology, which can be decreased by the influence of their close social network [25]. Various technology adoption studies have involved SI in theoretical frameworks and have confirmed SI as an essential antecedent in determining customers’ attitudes and behaviors [27,29,42,50]. Moreover, Chen and Lin (2018) claimed that the effect of SI was to dramatically formulate users’ mental awareness of engagement when using live-streaming [16]. Accordingly, interacting with relevant people on a specific information technology can facilitate users’ flow experience [23]. Hence, the current study proposes that users’ flow experience of LSSAs is positively determined by SI, which formulated the following hypothesis:

**Hypothesis** **4** **(H4).** *Social influence (SI) as a stimulus positively determines the organism flow (FL) when shopping via LSSAs during the pandemic lockdown period.*

Hedonic motivation (HM) was initially adapted in UTAUT2 by Venkatesh, Thong and Xu (2012), which is defined as the internal emotional perception of enjoyment and pleasure descend from a user’s expectation or experience of a certain information technology [13]. The directly positive effect of HM on adoption intention has been confirmed by prior researchers who applied UTAUT2 on mobile technology adoption, e.g., mobile payment [27] and mobile banking [26,28]. Meanwhile, HM has been recognised as an antecedent which also has a significant indirect effect on customers’ behavioral intention. Yeo, Goh and Rezaei (2017) claimed that HM formulated attitude via convenience motivation and post-usage usefulness when customers adopt online food delivery services [51]. Likewise, engagement, as the main characteristic of users’ flow experience, is formulated by enjoyment, curiosity and concentration [52,53].

Consequently, Wongkitrungrueng and Assarut (2018) validated HM directly and indirectly (through trust), formulating users’ engagement in live-streaming commerce [1]. Furthermore, Chen and Lin (2018) illustrated that live-streaming’s entertainment features formulated viewers’ HM, which positively determined their mental perception of value, which in turn affected their final behavioral intention [16]. Thus, the current study assumes that HM positively affects customers’ engagement and affection in Hypothesis 5.

**Hypothesis** **5** **(H5).** *Customers’ hedonic motivation (HM) as a stimulus positively determines the organism flow (FL) when shopping via LSSAs during the pandemic lockdown period.*

Gefen (2000) defined trust (TR) as describing users’ subjective awareness of believing a particular technology can fulfil obligations and positively guarantee a qualified performance to meet their expectations [54]. Specifically, under the lockdown measures of the COVID-19 pandemic, trust reflected users’ perceptions of technological characteristics such as mobility, security, etc., which correspond with perceived security against perceived risk and uncertainty conditions [44]. LSSAs’ contactless online consumption functions, a beneficial feature in the lockdown situation during the COVID-19 pandemic, formulated customers’ perceived trust and positively influenced enjoyable and practical cognitions [42]. Accordingly, trust as an essential variable in the investigation of users’ behavioral intention has been integrated into various adoption models, such as UTAUT2 [25], TAM [46] and the IS success model [37]. Moreover, from the participation aspect, the interaction between customers and vendors on live-streaming commerce platforms facilitated users’ perceived trust in sellers and products, which in turn optimized engagement [1]. Therefore, this paper proposes that trust formulates users’ engagement and cognitive acceptance, positively influencing flow [37,38]. Meanwhile, as an antecedent of users’ utilitarian perceptions, trust positively facilitates customers’ performance expectancy stimulus [26,46]. Hence, the following hypotheses are addressed:

**Hypothesis** **6** **(H6).** *Trust (TR) as a stimulus positively determines the organism flow (FL) when shopping via LSSAs during the pandemic lockdown period.*

**Hypothesis** **7** **(H7).** *Trust (TR) as a stimulus positively determines performance expectancy (PE) when shopping via LSSAs during the pandemic lockdown period.*

### 3.2. Organism Component: Flow (FL)

Csikszentmihalyi (1975) defined flow as an individual’s feeling of intrinsic absorption in a particular activity or technology [14]. Flow experience of technology was described as users’ temporary unawareness caused by their internal enjoyment of, pleasure in and engagement and interaction with a certain technology [16,38]. Moreover, lockdown measures provided an appropriate environment for the enhancement of an individual’s immersive shopping experience via LSSAs at home. Various technology adoption studies have validated the idea that flow is significantly formulated by users’ technological perceptions regarding their behavioral intention of adoption or continued usage [18,37,38]. Consequently, flow is in accordance with the conception of an organism, which is assumed as a mediator connecting technological and environmental stimuli and responses in shopping via LSSAs.

Flow has been examined by previous researchers as also having an indirect effect on users’ final responses, such as adoption intention, actual usage and continuance intention, via users’ mental reflection variables, such as perceived value, satisfaction and attitude [16,39,55]. Meanwhile, the effects of flow and mental reflections has been validated as a way in which to determine customers’ behavioral intentions conjointly in various pieces of literature [10,37,38]. Chen and Lin (2018) claimed that flow positively affected perceived value regarding the formulation of customers’ intention to use live-streaming [16]. Therefore, the following hypotheses are proposed:

**Hypothesis** **8** **(H8).** *Customers’ flow (FL) as an organism positively determines the response perceived value (PV) when shopping via LSSAs during the pandemic lockdown period.*

**Hypothesis** **9** **(H9).** *Customers’ flow (FL) as an organism positively determines the response behavioral intention (BI) when shopping via LSSAs during the pandemic lockdown period.*

### 3.3. Response Components: Perceived Value (PV) and Behavioral Intention (BI)

Perceived value (PV), as defined by Zeithaml (1988), represents customers’ universal assessments of a service or technology. PV is determined by users’ perceptions of acquisition and investment [56]. Sweeney and Soutar (2001) extended the dimensions of perceived value, including quality and price of production, customers’ emotional responses and social influence [57]. Meanwhile, Petrick (2002) modified behavioral price, monetary price, emotional response, quality and reputation, all of which emerged as other dimensions of PV [58]. PV also represents customers’ perceived multi-dimensional benefits, including those from utilitarian, hedonic and social perspectives [50,59]. Specifically, in this study, perceived value represents the customers’ general mental responses to shopping via LSSAs during the COVID-19 pandemic lockdown period. Perceived value has been assumed as a cognitive variable in various adoption models, such as the Expectation Confirmation Model [36], the Value-based Adoption Model [60] and the Mobile user Engagement Model [59], which positively determines customers’ behaviors. On the other hand, Chen and Lin (2018) confirmed that perceived value, as a conative factor, was determined by flow and, in turn, formulated users’ behaviors [16]. Therefore, this study proposes that perceived value, which, in a customer, would be a conational response, constitutes one component amongst a variety of responses, which is demonstrated in the following hypothesis:

**Hypothesis** **10** **(H10).** *Customers’ perceived value (PV) positively determines behavioral intention (BI) when shopping via LSSAs during the pandemic lockdown period.*

### 3.4. Moderation Hypotheses

Gender and age moderators were incorporated within the UTAUT2 model to investigate information technology adoption [13]. Various studies involved gender and age moderators in different adoption models, and validated gender and age as moderating constructs in different scenarios respectively [13,20,40,41,42,43,44,45]. As shown in Figure 1, gender and age moderators are assumed to be multi-group controls, which should optimize the predictive validity of the proposed model in the explanation of any conflicting results [42]. Meanwhile, due to the way in which the proposed model was initially developed, the moderation hypotheses are established by the exploratory approach in this study, which was common in previous studies [42]. The moderating effect will be assessed by subgroup analysis after the general model evaluation. Therefore, the following hypotheses are proposed in investigation of the different moderating effects of age and gender on customers’ psychological processes experienced while shopping via LSSAs.

**Hypothesis** **11** **(H11).** *Gender moderates the relations among all constructs of the proposed model.*

**Hypothesis** **12** **(H12).** *Age moderates the relations among all constructs of the proposed model.*

## 4. Methodology and Data Demographic Distribution

### 4.1. Measurement

A quantitative methodology was applied in this study to evaluate the proposed model. An online questionnaire survey was conducted to collect data in China, which comprised two parts. The first part requested demographic information (consisting of gender, age and frequency of using LSSAs during the COVID-19 pandemic lockdown period) of participants, using dichotomous, bounded continuous and ordinal-polytomous close-ended questions; the second part consisted of a seven-point Likert scale (from strongly disagree = “1” to strongly agree = “7”), comprising structural questions which evaluated performance expectancy (PE), effort expectancy (EE), social influence (SI), hedonic motivation (HM), trust (TR), flow (FL), perceived value (PV) and behavioral intention (BI), with 34 measurement items taken from previous literature, shown in Table A1 in Appendix A.

The questionnaire was designed and managed in English, before being translated into the Chinese language by language experts to avoid the biases of language and culture (the target population of the survey was smartphone users in China). Afterwards, according to the translation-back translation method, it was reverse-translated into the English language. In order to minimize the non-response rate, a short introduction and respondent-friendly survey questionnaire techniques were applied in the survey [61].

### 4.2. Data Collection

The online questionnaire was designed via Wenjuan.com (a Chinese online survey platform). According to the formulae from Westland (2010) and the numbers of 34 observed indicators and eight latent variables in the proposed model, the recommended minimum sample size for the model structure was 91 [62]. The questionnaires were distributed online and via WeChat (a Chinese mobile social media application) on 9 August 2020 for data collection. After four weeks of data collection, 400 empirical data were collected on 6 September 2020, of which 138 were derived from online responses and 262 via WeChat. After filtering out the responses with missing values in a scrutinizing process, 374 valid data were accepted for data analysis, which obtained a 93.5% final response rate. The Kolmogorov-Smirnov test was applied to examine the early respondents’ group with 100 participants and the late respondents with 274 participants. This test confirmed no statistical difference between the two independent groups [63]. Meanwhile, the Shapiro-Wilk test of the demographic data, age, gender and frequency of those shopping via LSSAs were 0.636, 0.862 and 0.918, respectively, and all showed a significant level of 0.000, which indicated the data was non-normally distributed.

### 4.3. Data Demographic Characteristics

The data was collected online and via WeChat randomly, and the geographical distribution of respondents consisted of 43.5%, 13.1% and 3.5% located in Henan, Guangdong and Shandong provinces, respectively, which are the three largest Chinese provinces in terms of population. This represented general smartphone users in China. 51.87% female and 48.13% male smartphone users participated in the survey. The largest age group was adults between 21 and 35, at 27.01%, while the age range of participants younger than 36 comprised 51.07%, with the group over 35 years old being 48.93%. These figures are consistent with the QusetMobile report (2020) that users between 19 and 35 years old were the leading group of shoppers via LSSAs in China [64]. Moreover, 24.06% of participants used LSSAs at least once per week. More than 39% of respondents shopped via LSSAs every week. Table 2 presents the specific demographic distribution of participants.

## 5. Data Analysis

As the proposed model was generated based on a solid theoretical foundation, structural equation modelling is appropriate to operationalizing the hypothesized latent constructs and associated indicators for theory development [65]. Besides minimizing the difference between the observed and estimated covariance matrices, covariance-based structural equation modelling (CB-SEM) applies a maximum likelihood procedure to assess correlations among all constructs and their interactive effects simultaneously [66,67]. Meanwhile, the proposed model in this study consists of mediating variables and moderators. The CB-SEM approach is well suited to the assessment of models involving mediation and moderating effects [65,67]. CB-SEM performs very accurately, with sum scores higher than both PLS-SEM and regression in a small sample size [68]. Meanwhile, CB-SEM presents more accurately than PLS-SEM for non-normally distributed data with a sample size over 50 [69]. CB-SEM provides optimal coefficient estimates and more accurate model analyses in the evaluation of research models [70]. Moreover, the two-step approach, consisting of the measurement model assessment and structural model evaluation [71], is applied in this study. Specifically, the CB-SEM technique is conducted for confirmatory factor analysis (CFA), to assess the convergent and discriminant validity for each construct in the measurement model assessment, and to evaluate the path coefficient to test hypotheses with a comparison of differences between age (<36 VS >35) and gender (Male VS Female) sub-samples in the structural model evaluation by AMOS.

Before implementing the two-step approach, The Exploratory Factor Analysis (EFA) was applied by SPSS to evaluate the dataset adequacy. The Kaiser criterion and scree plot were applied in order to identify the number of underlying extractive factors. The Kaiser test obtained an eight-factor solution with eigenvalues larger than 1, and the first inflexion point was located at the 9th point in the scree plot. The results indicate that eight factors can be extracted, which is aligned with the proposed model [72].

### 5.1. Measurement Model

Firstly, the reliability and validity of the measurement model was assessed by the following criteria: Construct reliability was confirmed by Cronbach’s alpha (CA). The CAs of all constructs exceeded 0.70 [73], and convergent validity was validated by factor loadings above 0.7 [74]; Composite Reliability (CR) exceeded 0.7; Average Variance Extracted (AVE) exceeded 0.5 [75]; discriminant validity was qualified by the square root of AVE of each latent construct exceeding any two pairs of its inter-construct correlation [75] and the AVE was higher than the maximum shared squared variance (MSV) of each construct [66]. The constructs’ CA, CR, AVE, MSV, and factor loading of items results are presented in Table 3.

Table 4 displays the results of the square root of AVE, which are bigger than the correlations of each latent construct. The values of the results reached the relevant recommended threshold of each criterion. Therefore, the reliability and validity of the measurement model were confirmed for further assessment.

Moreover, the model-fit of measurement model shown in Table 5 was validated by the goodness-of-fit results meeting the standards of each index [70]. Namely, the ratio of chi-square to degrees-of-freedom (X^2^/df < 3), comparative fit index (CFI > 0.9), the goodness of fit index (GFI > 0.8) [76], adjusted goodness-of-fit index (AGFI > 0.8) [70], normalized fit index (NFI > 0.9), Tucker-Lewis index (TLI > 0.9) and root mean square error of approximation (RMSEA < 0.08) [77].

Furthermore, the potential common method bias of this study was evaluated by Harman’s one-factor test and the fitness of a single-factor model. The result of Harman’s one-factor test is 49.63%, which meets the criteria proposed by Podsakoff et al. (2003) that the largest variance of one factor should be below 50% in order to confirm that a single factor cannot explain the majority of the variance [78]. The fitness results of a single-factor model are shown in Table 5, which illustrates the unqualified model fit of a single-factor model.

Based on the previous assessments, the measurement model is eligible for further structural model evaluation.

### 5.2. Structural Model

According to the research objectives and proposed hypotheses, the structural equation model was created by AMOS and developed into two versions with age and gender subgroups, respectively.

Firstly, the model-fits of structural models (including the original structural model and two structural models with age and gender subgroups) were assessed consistently as the previous evaluation process of model-fit of the measurement model. The results met all thresholds of goodness-of-fit as presented in Table 5, which demonstrates that all structural models have eligible goodness-of-fit.

Moreover, the R^2^ values of endogenous variables were assessed to evaluate the structural models’ explanatory powers. The R^2^ values of endogenous variables in the three structural models are presented in Table 6. Specifically, the model with gender subgroups has the highest R^2^ values of performance expectancy (R^2^ = 0.35), flow (R^2^ = 0.70) and behavioral intention (R^2^ = 0.45). The model with age subgroups has the highest R^2^ value of perceived value (R^2^ = 0.55).

Furthermore, the testing of the hypotheses was evaluated by the coefficient of each path. The results are depicted in Table 7. Specifically, except for H2 (EE → FL) being rejected (ß = 0.078, *p* = 0.068), all the other hypotheses were supported in the original structural model and sorted by the significance from high to low, shown as follows: H8 (FL → PV, ß = 0.732, *p* < 0.001), H9 (FL → BI, ß = 0.44, *p* < 0.001), H7 (TR → PE, ß = 0.38, *p* < 0.001), H10 (PV → BI, ß = 0.308, *p* < 0.001), H6 (TR → FL, ß = 0.275, *p* < 0.001), H5 (HM → FL, ß = 0.232, *p* < 0.001), H3 (EE → PE, ß = 0.209, *p* < 0.001), H4 (SI → FL, ß = 0.205, *p* < 0.001) and H1 (PE → FL, ß = 0.187, *p* < 0.001).

Moreover, based on the evaluation of path coefficients of each subgroup in Table 8, the model performed variously. With regards to moderation hypotheses, H11 and H12 were confirmed partially. Five out of ten paths were significantly different when comparing the gender groups. However, only one out of ten hypotheses were significantly different for age groups. The effect of flow on perceived values (H8) was validated, having the most significantly positive influence in all four subgroups. Meanwhile, the effect of flow on behavioral intention (H9) was verified with the second largest coefficient in the male subgroup (ß = 0.555, *p* < 0.001) and the over-35 subgroup (ß = 0.51, *p* < 0.001), respectively. The male subgroup significantly moderated effort expectancy on flow (ß = 0.162, *p* = 0.003). However, H2 (EE → FL) was rejected in the model with the female group (ß = 0.038, *p* = 0.55), the group with age below and equal to 35 (ß = 0.076, *p* = 0.18) and the group with age higher than 35 (ß = 0.084, *p* = 0.207). Meanwhile, relations between hedonic motivation and flow, and perceived value and behavioral intention, were significantly moderated by the female moderator. Hypotheses H5 (HM → FL, ß = 0.07, *p* = 0.248) and H10 (PV → BI, ß = 0.179, *p* = 0.083) were rejected in the model with the male subgroup. Likewise, the male subgroup moderated the effects of social influence and trust on flow. H4 (SI → FL, ß = 0.124, *p* = 0.062) and H6 (TR → FL, ß = 0.12, *p* = 0.155) were found with insignificant effects in the female subgroup. On the other hand, the age moderator only caused a significant difference in hedonic motivation as part of flow in two age groups, the younger age group having a more significant moderating effect on hedonic motivation as part of flow than the older age group.

In addition, the model invariances were evaluated by comparing the chi-square of two subgroup models to evaluate the moderating effects of gender and age, and to assess the H11 and H12; see the results presented in Table 9. H11 was supported by the results of the model, which demonstrated a variance under the moderating effect of gender. However, there were insignificant differences at the level of the model with different age groups, and thus H12 should be interpreted cautiously. Specifically, to illustrate the differences of the path effect in each subgroup, the critical ratio was assessed to test the hypotheses, and the z-score was tested to evaluate the data. As shown in Table 8, the results demonstrate the effects of hedonic motivation, trust and social influence on flow, as well as the effects of perceived values on behavioral intention, both of which were significantly variant between male and female groups. Meanwhile, only the flow path to perceived value significantly differed between younger and older age groups.

## 6. Discussion

Based on the key objectives of this research, as well as the data analysis results, the findings are discussed in the sequence of the stimulus-organism-response of customers’ psychological shopping processes via LSSAs under lockdown measures during the COVID-19 pandemic. Specifically, the significant determinants of each subgroup’s stimulus-organism-response components were summarized in Table 10.

The variables from the revised UTAUT2 model, as the stimulus in users’ psychological processing, demonstrated variance in different sub-models. Specifically, except for performance expectancy, which had significant effects on flow in all subgroups, the other path effects of antecedences of flow presented differently in different subgroups. Effort expectancy only significantly affected flow in the male subgroup, contrary to the previous findings [45]. This study found that male customers’ engagement and immersion were more determined regarding the understandability, accessibility and operability of LSSAs. On the other hand, effort expectancy had a positive influence on performance expectancy in all subgroups, which was consistent with previous findings that when customers perceive the ease of using LSSAs, they will feel using an LSSA is a useful and efficient way to shop online [26,43,79]. LSSA providers should maintain applications with easily understandable interfaces and functions to increase the accessibility of LSSAs.

Meanwhile, social influence had a significant effect on flow in all subgroups except for female customers, which was consistent with the findings of Liébana-Cabanillas, Sánchez-Fernández and Muñoz-Leiva [41], but contrary to the results of Pascual-Miguel, Agudo-Peregrina and Chaparro-Peláez’s study [45]. The results of this study validated that female customers are more influenced with more difficulty by other relevant people when they purchase through LSSAs during the COVID-19 pandemic lockdown period. However, recommendations and support from relevant important people significantly formulate users’ mental cognition in the male subgroup and both age subgroups, which means they would feel less anxiety and uncertainty if provided with the support of important, relevant people when using LSSAs during the pandemic lockdown period [25,80]. When customers’ close friends or families are engaged in LSSAs, they are more inclined to participate and interact with sellers on LSSAs [16]. Therefore, word-of-mouth marketing is an efficient and reliable way to establish the excellent reputation of LSSAs, to increase male customers’ engagement and to increase enjoyment when shopping via LSSAs during the pandemic lockdown period.

Moreover, hedonic motivation had more significant influences on the younger female group when they were shopping via LSSAs during the pandemic lockdown period. Therefore, the enjoyment of live-streaming content, as well as its entertainment features, are essential to optimize users’ experience and increase engagement, especially for younger female customers. Furthermore, trust had a significant impact on flow in all age subgroups and the male group. Specifically, male customers paid more attention to the trustworthiness and security of LSSAs [44]. On the other hand, trust was an essential antecedent of flow in all age ranges, which was opposite to the findings of Liébana-Cabanillas, Sánchez-Fernández and Muñoz-Leiva (2014), where trust was more affected by younger groups [41]. Under the situation of social commerce lockdown, live-streaming production demonstration increased customers’ perceived trust by providing reliable control, which positively influenced consumers’ shopping experiences during the pandemic lockdown period [38]. Likewise, trust had a significant influence on performance expectancy in all subgroups. When users perceive a higher sense of trust in shopping via LSSAs under the pandemic lockdown measures, their holistic mental perceptions of the utility of technology will increase accordingly [80]. Therefore, information accuracy, information security and customers’ privacy should be guaranteed by LSSA providers, especially for male customers [39].

Furthermore, flow, as an organism of users’ psychological processing, had the most significant effects on perceived value and behavioral intention in all subgroup models, which is consistent with previous findings, which suggest that engagement and an immersive experience can significantly formulate users’ mental and physical reactions to shopping via LSSAs during the pandemic lockdown period [16,38,39]. Specifically, flow as mediator in the proposed model performed diversely in each subgroup, the proportion of the variance for flow in gender subgroup model and age subgroup model being 0.70 and 0.58, respectively, which demonstrates the higher explanatory power in the model with the gender subgroup than the model with the age subgroup. Meanwhile, comparing the coefficients of flow with other variables between four subgroup models, flow has the most significant relations with effort expectancy, social influence, trust and behavioral intention in the male subgroup, while flow has the strongest relations with hedonic motivation and perceived value in the female group. Performance expectancy has the most significant effect on flow in the young age subgroup. The result indicates that functional and environmental factors affect young male customers’ immersive experience more, while the entertainment factor influences female users’ flow experience more. When customers are immersed in live-streaming shopping, they tend to escape from the pandemic situation and forget about time and problems, which irrelevant things do not easily disturb [10]. Therefore, optimizing customer engagement and interaction in live-streaming demonstrations is necessary to increase users’ immersive shopping experiences via LSSAs in the pandemic lockdown situation [38].

In addition, in response components, perceived value significantly determined customers’ behavioral intention regarding the use of LSSAs in all subgroups except male customers. When customers feel pleasure when purchasing through LSSAs during the pandemic lockdown period, they will perceive higher multi-dimensional benefits of LSSAs, including utilitarian, hedonic and social benefits, which in turn significantly determine customers’ usage intention [16,50,59]. Male customers’ holistic perceptions of the benefits of LSSAs might be indirectly influenced by their perceptions of technology [9]. Therefore, it is necessary to optimize the interfaces and functions of LSSA-services for improving the practicability, usability and creditability of LSSAs, to attract male customers’ engagement. These methods can increase users’ enjoyment, recognition and satisfaction when shopping through LSSAs, contributing towards formulating their actual usages.

## 7. Theoretical and Practical Implications

### 7.1. Theoretical Implications

As shopping via live-streaming is becoming an immensely popular social and commercial phenomenon, the factors determining customers’ intentions regarding shopping via live-streaming apps have attracted increasing attention in recent years. Current research has demonstrated novel insights into explaining customers’ psychological shopping processes via LSSAs during the COVID-19 pandemic lockdown period. This study contributes three theoretical implications. Firstly, this study bridges a gap in the existing literature by initially evaluating the moderating effects of gender and age on the determinants of customers’ psychological processing in the use of LSSAs, which enriches the literature of relevant fields and verifies previous findings regarding the moderating effects of gender and age. Comparing the influences of different moderators, especially gender and age, on each path provides a better understanding of the effects of customers’ demographic characteristics on LSSA adoption. Secondly, this study contributes to theoretical development by extending the SOR framework with UTAUT2 and Flow Theory. Notably, this study integrates the variables from the revised UTAUT2 model as the stimulus component of the SOR framework, and Flow Theory supports the organism in the SOR framework as a mediator of the adoption model. The comprehensive model was validated in this study to support the understanding of applying the SOR framework in the LSSA adoption context. Thirdly, the current study successfully explains that customers’ psychological processes experienced while shopping via LSSAs under the pandemic lockdown condition are induced by perceived technological perceptions (performance expectancy, effort expectance, hedonic motivation) and environmental perceptions (social influence, trust), mediated by mental cognition (flow) and demonstrated by actual responses (perceived value and behavioral intention). This finding generates new insights for future research to assess various connections, interactions, and relationships among the variables between or within different components in the SOR framework for different technology adoption studies.

### 7.2. Practical Implications

This study’s results are essential for LSSA-service providers, LSSA sellers, streamers and relevant stakeholders interested in the live-streaming commerce industry. The current study supports stakeholders relevant to LSSAs in understanding the behaviors of different customer demographics influenced by the moderating effects of gender and age. In particular, hedonic motivation, trust and social influence had the most significant differences in male and female groups. This study provides insights for LSSA stakeholders, encouraging them to consider gender differences affecting various antecedents at different stages of psychological processes experienced by customers while shopping via LSSAs, helping them to create or manage a better strategy for their target customers in the future. For example, LSSA providers and vendors should focus on maintaining a relaxing and comfortable live-streaming environment and guaranteeing the originality and fascination of the live-streaming context to optimize entertainment for attracting female customers.

Moreover, this study helps LSSA-platform providers, streamers and LSSA sellers, acting as a guidebook to understanding each component in the mental processes undergone by customer when using LSSAs for shopping during the pandemic lockdown period. Based on the findings, flow had both significant effects on perceived value and behavioral intention. LSSA-platform providers should emphasize user-centered principles to guarantee the reliability, convenience and efficiency of LSSA-services to meet customers’ expectations and requirements, helping to formulate an immersive environment for customers to improve their engagement and optimize their mental cognition of shopping through LSSAs. Streamers and LSSA sellers should ensure entertainment, instantaneity and accuracy of interactions with customers to formulate a pleasant and enjoyable environment for optimizing their shopping experience when using LSSAs. Furthermore, the current research contributes a framework for the investigation of customers’ mental processes under a specific environmental condition. This study proposed a critical procedure (technological and environmental perceptions → engagement and mental cognition → reaction) to evaluate customers’ psychological processes. Meanwhile, the assessment and evaluation of the moderating effects of gender and age applied in this study provide a reference with which to analyze demographically different customers’ behaviors. Relevant stakeholders can generate particular strategies for their different customers based on the variation of the moderating effects of gender and age on different determinants.

## 8. Conclusions

Shopping via live-streaming is booming after the lockdown measures of the COVID-19 pandemic. This study investigated the psychological processes undergone by customer shopping via LSSAs during the COVID-19 pandemic lockdown period in China. The proposed model extended the SOR framework with UTUAT2 and Flow Theory and was tested by CB-SEM with 374 valid data, with four subgroups divided by age and gender.

### 8.1. Result of the Study

The empirical results demonstrate that flow as a mediator had the most significant influence on users’ responses. Technological and environmental perceptions significantly formulate customers’ engagement and immersive experience, which determine their behaviors. This study validates that gender has significant moderating effects on effect expectancy, hedonic motivation, trust, social influence and perceived value. Specifically, effect expectancy, social influence and trust had significant effects on flow in the male group. On the other hand, hedonic motivation and perceived value were found to significantly affect female customers’ psychological processes when shopping via LSSAs. Moreover, hedonic motivation has a more significant effect on flow in younger customers than in older customers.

The current research provides a better understanding of customers’ psychological processes under a particular condition, namely, the COVID-19 lockdown situation. The current study contributes a theoretical development, helping to integrate psychological framework with technological adoption models, and provides a practical guideline on investigating the psychological processes experienced by customer who shopped via LSSAs under the pandemic lockdown situation. This helps by supporting relevant researchers and stakeholders in understanding customers’ behaviors under a specific condition.

### 8.2. Limitations and Future Research

Although the current study proposed a rigorous framework of psychological processing on behalf of customers to adopt LSSAs, four limitations are summarized as follows with correspondent recommendations for future research. First, this study’s target location was China, which indicates the limited generalizability of results in different cultures, regions and countries. Therefore, future researchers are recommended to pay more attention to investigating relevant studies in various regions and cultural backgrounds, as well as to make comparisons between locations with different cultures. Second, moderators analyzed in this study only consisted of the participants’ basic demographic characteristics, namely, gender and age. Various moderators can contribute different moderating effects to different constructs in the model. Thus, future research is recommended to investigate users’ behaviors under various moderating effects, such as experience, educational background, Hofstede’s cultural values, etc. Third, this research did not distinguish the types of LSSAs in the study. The different types of LSSAs may lead to different results [6].

Consequently, future research is recommended to distinguish the differences between various technologies and platforms. Last, this study conducted a four-week data collection during the COVID-19 pandemic lockdown period, indicating the limitation of a short investigation period for generalizing an overall analysis in different scenarios. Thus, a long-term approach, as well as a comparison between customers’ different stages of shopping experiences via LSSAs and under different situations might be several meritorious directions for future research.

## Figures and Tables

**Table 1:** Literature review of the SOR framework.

Studies	Topic	Stimulus	Organism	Response
[22]	Mobile shopping	Ubiquity;	Impulsive buying tendency;Normative evaluation; Positive affect	Purchase intention
		Ease of use;		
		Information exchange;		
		Discounted price;		
		Scarcity		
[11]	Social commerce	Structural capital;	Consumer value	Consumer loyalty
		Cognitive capital;		
		Relational capital;		
		Social identification;		
		Social influence;		
		Social commerce needs;		
		Social commerce risk;		
		Social commerce convivence;		
[10]	Mobile payment	Usefulness;	Flow	Satisfaction; Purchase intention
		Emotion;		
		Security		
[12]	Virtual reality tourism	Actual experiences	Enjoyment,	Attachment; Visit intention
			Emotional involvement,	
			Flow	
[23]	Massive open online courses	Interactivity;	Virtual Experience; Telepresence; Social presence; flow	Continuance intention
		Media richness;		
		Sociability		

**Table 2:** Demographic distribution of participants.

Measure	Item	*N*	%
Gender	Male	180	48.13%
	Female	194	51.87%
Age	<20	90	24.06%
	21–35	101	27.01%
	36–50	96	25.67%
	>51	87	23.26%
Frequency of using LSSAs during the COVID-19 pandemic lockdown period	At least 1 time per 1 day	59	15.78%
	At least 1 time per 1 week	90	24.06%
	At least 1 time per 2 weeks	81	21.66%
	At least 1 time per 1 month	55	14.71%
	At least 1 time per 3 months	41	10.96%
	At least 1 time per 6 months	29	7.75%
	Never used during the pandemic lockdown period	19	5.08%

**Table 3:** Latent constructs’ CA, CR, AVE, MSV, and items’ factor loading.

Factors	CA	CR	AVE	MSV	Items	Loadings
Performance expectancy (PE)	0.943	0.943	0.807	0.510	PE1	0.875
					PE2	0.912
					PE3	0.905
					PE4	0.899
Effort expectancy (EE)	0.935	0.932	0.820	0.326	EE1	0.896
					EE2	0.874
					EE3	0.916
					EE4	0.858
Social influence (SI)	0.949	0.936	0.785	0.264	SI1	0.896
					SI2	0.904
					SI3	0.925
					SI4	0.902
Hedonic motivation (HM)	0.946	0.949	0.822	0.416	HM1	0.868
					HM2	0.89
					HM3	0.909
					HM4	0.869
					HM5	0.869
Trust (TR)	0.948	0.946	0.776	0.412	TR1	0.905
					TR2	0.883
					TR3	0.873
					TR4	0.898
					TR5	0.873
Flow (FL)	0.965	0.948	0.786	0.446	FL1	0.911
					FL2	0.923
					FL3	0.930
					FL4	0.910
					FL5	0.927
Perceived value (PV)	0.943	0.965	0.847	0.510	PV1	0.882
					PV2	0.901
					PV3	0.909
					PV4	0.900
Behavioral Intention (BI)	0.923	0.923	0.800	0.394	BI1	0.892
					BI2	0.892
					BI3	0.899

(CA = Cronbach’s alpha; CR = Composite Reliability; AVE = Average Variance Extracted; MSV = maximum shared squared variance).

**Table 4:** Latent constructs’ square root of AVE and correlation.

	PV	PE	EE	SI	HM	TR	FL	BI
**PV**	**0.898**							
**PE**	0.571	**0.905**						
**EE**	0.514	0.416	**0.886**					
**SI**	0.645	0.408	0.427	**0.907**				
**HM**	0.642	0.422	0.463	0.551	**0.881**			
**TR**	0.668	0.506	0.460	0.565	0.535	**0.886**		
**FL**	0.714	0.523	0.470	0.599	0.592	0.635	**0.920**	
**BI**	0.608	0.494	0.486	0.538	0.541	0.600	0.628	**0.894**

(Number in **Bold:** Latent constructs’ square root of AVE).

**Table 5:** The Model-fit of each model.

	X^2^/df	CFI	GFI	AGFI	NFI	TLI	RMSEA
Recommend Value	<3	>0.9	>0.8	>0.8	>0.9	>0.9	<0.08
Single-Factor Model	12.471	0.526	0.398	0.360	0.505	0.525	0.175
Measurement Model	1.166	0.994	0.918	0.902	0.959	0.993	0.021
Original Structural Model	1.477	0.982	0.899	0.882	0.947	0.980	0.036
Model with Age Subgroups	1.433	0.968	0.825	0.796	0.902	0.965	0.034
Model with Gender Subgroups	1.371	0.972	0.829	0.801	0.906	0.970	0.032

**Table 6:** R^2^ values of endogenous variables in different models.

Endogenous Variables	R^2^		
	Original Structural Model	Model with Age Subgroups	Model with Gender Subgroups
**PE**	0.30	0.30	0.35
**FL**	0.58	0.58	0.70
**PV**	0.53	0.55	0.52
**BI**	0.45	0.42	0.45

**Table 7:** Hypotheses testing of the original structural model.

		Original Model				
**H**	**Relations**	**Estimate**	**S.E.**	**T**	**P**	**Decisions**
**H1**	PE→FL	0.187	0.047	3.958	***	Supported
**H2**	EE→FL	0.078	0.043	1.825	0.068	Rejected
**H3**	EE→PE	0.209	0.048	4.319	***	Supported
**H4**	SI→FL	0.205	0.044	4.642	***	Supported
**H5**	HM→FL	0.232	0.052	4.485	***	Supported
**H6**	TR→FL	0.275	0.053	5.196	***	Supported
**H7**	TR→PE	0.38	0.052	7.323	***	Supported
**H8**	FL→PV	0.732	0.046	15.799	***	Supported
**H9**	FL→BI	0.44	0.069	6.38	***	Supported
**H10**	PV→BI	0.308	0.069	4.469	***	Supported

(Est. = estimate; S.E. = standard error; T = t-value; P = *p*-value; ***: *p*-value < 0.01).

**Table 8:** Hypotheses testing of the subgroups.

		Model with Gender Subgroups										
		Male					Female					
**H**	**Relations**	**Est.**	**S.E.**	**T**	**P**	**Dec.**	**Est.**	**S.E.**	**T**	**P**	**Dec.**	**Z-Score**
**H1**	PE→FL	0.146	0.061	2.401	0.016	Sup.	0.186	0.068	2.73	0.006	Sup.	0.446
**H2**	EE→FL	0.162	0.054	3.014	0.003	Sup.	0.038	0.064	0.598	0.55	Rej.	−1.485
**H3**	EE→PE	0.265	0.066	4.009	***	Sup.	0.151	0.07	2.151	0.031	Sup.	−1.173
**H4**	SI→FL	0.283	0.056	5.075	***	Sup.	0.124	0.067	1.866	0.062	Rej	−1.83 *
**H5**	HM→FL	0.07	0.061	1.154	0.248	Rej.	0.465	0.088	5.297	***	Sup.	3.699 ***
**H6**	TR→FL	0.367	0.064	5.697	***	Sup.	0.12	0.084	1.421	0.155	Rej.	−2.331 **
**H7**	TR→PE	0.385	0.071	5.447	***	Sup.	0.389	0.076	5.145	***	Sup.	0.035
**H8**	FL→PV	0.681	0.066	10.293	***	Sup.	0.771	0.065	11.902	***	Sup.	0.973
**H9**	FL→BI	0.555	0.099	5.609	***	Sup.	0.337	0.096	3.526	***	Sup.	−1.583
**H10**	PV→BI	0.179	0.103	1.734	0.083	Rej.	0.409	0.092	4.46	***	Sup.	1.664 *
		**Model with Age Subgroups**										
		**≤35**					**>35**					
**H**	**Relations**	**Est.**	**S.E.**	**T**	**P**	**Dec.**	**Est.**	**S.E.**	**T**	**P**	**Dec.**	**Z-Score**
**H1**	PE→FL	0.196	0.063	3.106	0.002	Sup.	0.182	0.071	2.57	0.01	Sup.	1.431
**H2**	EE→FL	0.076	0.056	1.341	0.18	Rej.	0.084	0.067	1.263	0.207	Rej.	−0.153
**H3**	EE→PE	0.145	0.068	2.131	0.033	Sup.	0.285	0.07	4.097	***	Sup.	−0.411
**H4**	SI→FL	0.21	0.063	3.319	***	Sup.	0.205	0.062	3.295	***	Sup.	0.096
**H5**	HM→FL	0.287	0.066	4.383	***	Sup.	0.155	0.082	1.892	0.058	Rej	−0.058
**H6**	TR→FL	0.204	0.076	2.666	0.008	Sup.	0.342	0.075	4.58	***	Sup.	−1.259
**H7**	TR→PE	0.479	0.074	6.512	***	Sup.	0.277	0.073	3.773	***	Sup.	1.293
**H8**	FL→PV	0.744	0.065	11.37	***	Sup.	0.72	0.065	10.995	***	Sup.	−1.947 *
**H9**	FL→BI	0.352	0.099	3.566	***	Sup.	0.51	0.096	5.308	***	Sup.	−0.268
**H10**	PV→BI	0.341	0.1	3.405	***	Sup.	0.284	0.095	2.983	0.003	Sup.	1.15

(Est. = estimate; S.E. = standard error; T = t-value; P = *p*-value; Dec.= decision; Sup. = Supported; Rej. = Rejected; ***: *p*-value < 0.01; **: *p*-value < 0.05; *: *p*-value < 0.1).

**Table 9:** Comparison between the models of gender and age subgroups.

	Model with Gender Subgroups			Model with Age Subgroups		
	Chi-Square	df	*p*-Value	Chi-Square	df	*p*-Value
Unconstrained	1401.159	1022		1464.315	1022	
Fully Constrained	1451.548	1058		1496.514	1058	
Number of Groups	2			2		
Difference	50.389	36	0.056	32.199	36	0.650
Model Invariant	NO			YES		

**Table 10:** The significant determinants of each subgroup.

Mediator	Subgroup	Stimulus	Organism	Response
Gender	Male	Performance expectancy;	Flow	Behavioral Intention
		Effort expectancy;		
		Social influence;		
		Trust		
	Female	Performance expectancy;	Flow	Perceived value;
		Hedonic motivation;		Behavioral Intention
Age	≤35	Performance expectancy;	Flow	Perceived value; Behavioral Intention
		Social influence;		
		Hedonic motivation;		
		Trust		
	>35	Performance expectancy;	Flow	Perceived value; Behavioral Intention
		Social influence;		
		Trust		

## Data Availability

Data available on request due to restrictions eg privacy or ethical.

## Appendix A

**Table A1:** Online questionnaire.

Dear participant: Thank you for taking a few minutes to participate in this questionnaire! The purpose of this questionnaire is to collect the factors of shopping via live-streaming shopping app. (i.e., Douyin app, Kuaishou app, Taobao live, etc.). The questionnaire is anonymous. The data obtained is only for academic research. Please feel free to fill in the questionnaire according to your personal situation.		
Thank you for your support!		
**Part 1–Demographic Information**		
**Measure**	**Item**	
Gender	Male	
	Female	
Age	<20	
	21–35	
	36–50	
	>51	
Frequency of using LSSAs during lockdown period	At least 1 time per 1 day	
	At least 1 time per 1 week	
	At least 1 time per 2 weeks	
	At least 1 time per 1 month	
	At least 1 time per 3 months	
	At least 1 time per 6 months	
	Never used during lockdown period	
**Part 2–Structural Evaluation**		
**Construct**	**Items**	**References**
Performance expectancy (PE)	PE1: I feel using LSSA is a useful way of shopping during lockdown period.	[13]
	PE2: Using LSSAs makes purchasing easier during lockdown period.	
	PE3: Using LSSAs improves my shopping efficiency during lockdown period.	
	PE4: Using LSSAs makes shopping more convenient during lockdown period.	
Effort expectancy (EE)	EE1: Learning how to use LSSAs is easy.	[13]
	EE2: It is easy to follow all the functions of LSSAs.	
	EE3: It is easy to become skillful at using LSSAs.	
	EE4: Interaction with LSSAs is clear and comprehensible.	
Social influence (SI)	SI1: People who are important to me (e.g., family members, close friends, and colleagues) recommend I use LSSAs for shopping during lockdown period.	[13]
	SI2: People who are important to me view LSSA as beneficial way for shopping during lockdown period.	
	SI3: People who are important to me think it is a good idea to use LSSAs for shopping during lockdown period.	
	SI4: People who are important to me support my use of LSSAs.	
Hedonic motivation (HM)	HM1: Shopping via LSSAs is entertaining during lockdown period.	[1,13]
	HM2: Shopping via LSSAs relaxes me during lockdown period.	
	HM3: Shopping via LSSAs gives me pleasure during lockdown period.	
	HM4: Activities (e.g., flash sales, freebies) on LSSAs make me excited.	
	HM5: I enjoy shopping via LSSAs during lockdown period.	
Trust (TR)	TR1: I believe LSSAs are competent and effective in handling customers’ shopping activities.	[1,13]
	TR2: I believe LSSAs keep customers’ interests in mind.	
	TR3: I trust the product demonstration from high-reputation sellers on LSSAs.	
	TR4: I believe that the products I purchase from LSSAs will be the same as those demonstrated on LSSAs.	
	TR5: Overall, I believe LSSAs are trustworthy way for shopping during lockdown period.	
Flow (FL)	FL1: When using LSSAs, my attention is focused on the shopping activities.	[16,38]
	FL2: When shopping via LSSAs, I do not realize how time passes.	
	FL3: Using LSSAs gives me a temporary escape from the real-world pandemic situation.	
	FL4: While shopping through LSSAs, I am able to forget my problems.	
	FL5: When shopping via LSSAs, I often forget the work I should do.	
Perceived value (PV)	PV1: Using LSSAs makes shopping more efficient and safer during lockdown period.	[50]
	PV2: Shopping via LSSAs would allow me to take advantage of additional promotions during live-streaming.	
	PV3: Shopping via LSSAs provides me with a lot of enjoyment, or gives me happiness during lockdown period.	
	PV4: Given the time I need to spend doing it during lockdown period, shopping via LSSAs is worthwhile to me.	
Behavioral intention (BI)	BI1: Shopping via LSSAs had become one of consumption and entertainment patterns for me.	[13]
	BI2: Given the opportunity, I will continuously shop via LSSAs in future.	
	BI3: I would like to recommend others to use LSSAs for shopping during lockdown period.	
